# MLKL: Functions beyond serving as the Executioner of Necroptosis

**DOI:** 10.7150/thno.54072

**Published:** 2021-03-04

**Authors:** Chaoning Zhan, Minchun Huang, Xiaojun Yang, Jin Hou

**Affiliations:** Department of Stomatology, Nanfang Hospital, Southern Medical University, Guangzhou, P.R. China.

**Keywords:** MLKL, necroptosis, structure, inhibitors, therapeutic target.

## Abstract

Recently, necroptosis, as a programmed cell death pathway, has drawn much attention as it has been implicated in multiple pathologies, especially in the field of inflammatory diseases. Pseudokinase mixed lineage kinase domain-like protein (MLKL) serves as a terminal-known obligate effector in the process of necroptosis. To date, the majority of research on MLKL has focused on its role in necroptosis, and the prevailing view has been that the sole function of MLKL is to mediate necroptosis. However, increasing evidence indicates that MLKL can serve as a regulator of many diseases via its non-necroptotic functions. These functions of MLKL shed light on its functional complexity and diversity. In this review, we briefly introduce the current state of knowledge regarding the structure of MLKL, necroptosis signaling, as well as cross-linkages among necroptosis and other regulated cell death pathways, and we particularly highlight recent progress related to newly identified functions and inhibitors of MLKL. These discussions promote a better understanding of the role of MLKL in diseases, which will foster efforts to pharmacologically target this molecule in clinical treatments.

## Introduction

Cell death is an irreversible degeneration process that results in the loss of cell integrity and is crucial for organ development, tissue homeostasis, and host defense 1, 2. There are two types of cell death, namely accidental cell death (ACD) and regulated cell death (RCD). While the former is unpreventable, the latter can be modulated by pharmacologic and/or genetic interventions 3. In 1972, the caspase-dependent apoptosis pathway was the first type of RCD morphotype to be discovered 4, and our understanding of its molecular mechanism has grown considerably. However, only in the past decade has caspase-independent programmed necrosis or 'necroptosis' started to be a major focus in efforts to understand cell death and the pathology of inflammatory diseases. A key finding has been that cell fate can be determined by a switch from apoptosis to necroptosis via genetic ablation of caspase 8 (CASP8) or by using caspase inhibitors. In the natural state, if pathogens inhibit CASP8 to evade host defense, they will encounter the second guard pathway, namely necroptosis 5. Although necroptosis may be a backup mechanism to clear pathogens, it can also trigger a strong inflammatory response, which substantiates the detrimental side-effect of necroptosis 6.

Because apoptosis is indispensable for mammalian development 7, 8, apoptosis is not an ideal target for controlling inflammation. Thus, its counterpart defense mechanism, necroptosis, may represent an attractive target goal. Studies have shown that mixed lineage kinase domain-like protein (MLKL)-deficient mice are viable, healthy, and fertile and do not display any physical or behavioral abnormalities 9, 10. Thus, MLKL, acting as the most terminal obligate effector in necroptosis seems to be a desirable target for drug development 10. Moreover, the traditional view suggests that the only function of MLKL is compromising cellular integrity, thus resulting in necroptosis, which would limit the side effects of MLKL inhibitors. However, more recent studies have found that in addition to its well-known role in necroptosis, MLKL has been found to play regulatory roles in a number of other physiological and pathophysiological events. These beneficial non-necroptotic functions of MLKL, such as limiting intracellular bacteria replication and promoting nerve regeneration 11, 12, would likely be affected by MLKL-targeting drugs aimed at controlling inflammation. Besides, a clear understanding of the cellular and molecular mechanisms of the newly discovered functions of MLKL is also needed to aid the development of new treatment strategies for relevant diseases, such as multiple sclerosis and cancer 13, 14. Based on the existing studies, the non-necroptotic functions of MLKL are summarized for the purpose of sharing a contemporary understanding of MLKL-based biology.

## Structure of MLKL

More than 500 protein kinases have been identified in the human genome, with approximately 10% of them appearing to be enzymatically inactive and having been classified as pseudokinases 15. The primary function of protein kinases is to phosphorylate protein substrates, which is important in cellular homeostasis. In general terms, protein kinases rely on the ATP interactor 'VAIK' motif, the catalytic loop 'HRD' motif, and the metal co-factor binding 'DFG' motif 10. All of these are crucial for phosphoryl transfer activity. However, the 'HRD' motif and 'DFG' motif are defective or absent in MLKL orthologs 16. Thus, MLKL is classified as a pseudokinase based on its lack of two of the three conserved catalytic residues in its kinase-like domain 15. Generally, divalent metals, most typically of Mg^2+^, can neutralize the net negative charge of the nucleotide and enable the kinase active site to interact with nucleotides. Intriguingly, MLKL can bind nucleotides in the absence of cations 10, 17.

**Figure 1:**
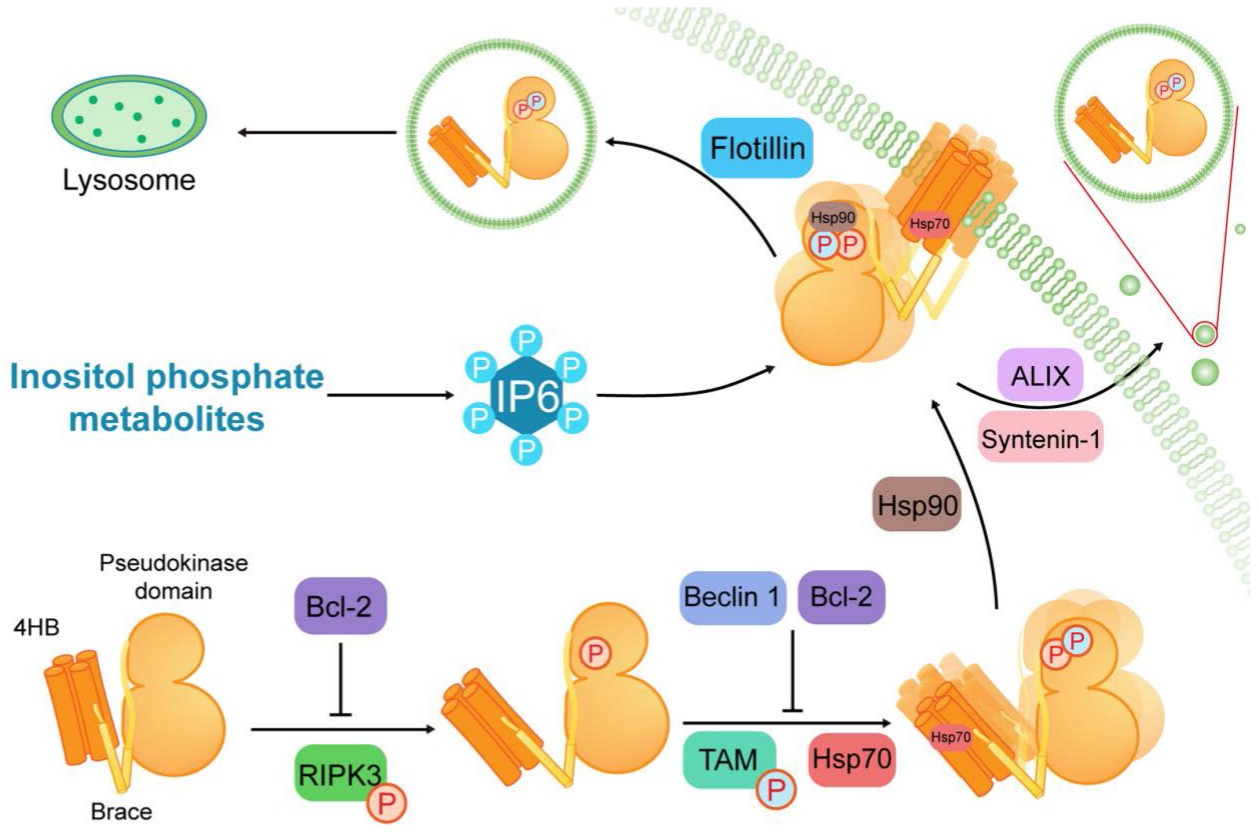
** Structure of MLKL and its regulation in necroptosis.** MLKL contains a C-terminal pseudokinase domain, a two-helix brace and an N-terminal four-helix bundle (4HB). After MLKL is phosphorylated by RIPK3, TAM (Tyro3, Axl, and Mer) kinase phosphorylates MLKL at the pseudokinase domain to initiate oligomerization of MLKL. Then MLKL is conjugated to heat shock protein 90 (Hsp90) and Hsp70 to achieve membrane translocation. Furthermore, inositol phosphate metabolites are required for the IP_6_ production. And IP_4_, IP_5_ and IP_6_ serve as critical binders of MLKL in necroptosis. During the course of MLKL activation, Beclin 1 and B-cell lymphoma 2 (Bcl-2) play negative regulatory roles in the conformation and oligomerization of MLKL. Additionally, flotillin-mediated endocytosis and ALIX-syntenin-1-mediated exocytosis can preclude contact between MLKL and the plasma membrane to suppresses necroptosis.

MLKL, as a pseudokinase, consists of a C-terminal pseudokinase domain, a two-helix brace or linker, and an N-terminal four-helix bundle (4HB) (Figure 1) 10, 18. The phosphorylation sites of RIPK3 exist on the MLKL activation loop residues, mouse serine 345 16, 19 or human threonine 357/serine 358 20, in the pseudokinase domain. Upon phosphorylation of MLKL, the conformation of the pseudokinase domain will change, which results in 4HB domain exposure 16, 21, 22. Additionally, there are other phosphorylation events that modulate MLKL function. Mouse serine 158 is a MLKL phosphorylation site that is subject to as-yet-unknown protein-mediated phosphorylation, and its phosphorylation helps to suppress cell death 23.

The N-terminal region of MLKL consists of a 4HB domain interacting with a two-helix brace 10. Generally, MLKL disulfide bond-dependent oligomerization and membrane translocation are essential for the formation of membrane pores 21, 22, 24-27. The oligomerization is initiated by TAM (Tyro3, Axl, and Mer) kinase, which can phosphorylate MLKL at tyrosine 376 28. The membrane translocation is based on patches of positively charged amino acids that are localized over the surface of the 4HB, which can interact with phosphatidylinositol phosphates (PIPs) 29, 30. This process requires several inositol phosphate (IP) (e.g., IP_4_, IP_5_ and IP_6)_ and inositol pentakisphosphate 2-kinase, which transforms IP_5_ into IP_6_ 31. Conversely, flotillin-mediated endocytosis and ALIX-syntenin-1-mediated exocytosis can prevent contact between phosphorylated MLKL (p-MLKL) and the plasma membrane to inhibit necroptosis 32. As a consequence, oligomerization and membrane translocation are subject to complex multilevel regulation to prevent these processes from getting out of control.

The late formation of small pores around 4 nm in diameter is a core event in necroptosis 33. Furthermore, MLKL-induced cell death is positively correlated with the activity of cation channels formed by MLKL 34. In addition to phosphatidylinositol phosphates (e.g., PI(5)P and PI(4,5)P_2_) within plasma membranes 29, 35, cardiolipin can also bind MLKL oligomer 36. Cardiolipin is mainly distributed in the mitochondrial inner membrane. Although several studies have implicated mitochondria and mitochondrial reactive oxygen species (ROS) production as being involved in necroptosis 37-39, specific removal of mitochondria does not compromise necroptosis 40.

It is significant to note that MLKL can act as a client protein in interacting with different molecular chaperone proteins. Heat shock protein 90 (Hsp90) binds the pseudokinase domain of MLKL via its cochaperone CDC37. This weak or transient interaction is required for MLKL function and may be involved in the phosphorylation of MLKL 41. Another molecular chaperone, heat shock protein 70 (Hsp70), can stabilize MLKL and promote MLKL polymerization via binding to the N-terminal region of MLKL 42. Such evidence demonstrates that the molecular chaperone proteins can promote necroptosis via interacting with different sites in the MLKL structure. In contrast, some proteins function as negative modulators of MLKL. For example, Beclin 1 and B-cell lymphoma 2 (Bcl-2) can bind to the coiled-coil domain and Bcl-2 homology (BH)-3 domain of MLKL, respectively, to inhibit necroptosis 43, 44.

## Necroptosis signaling

**Figure 2:**
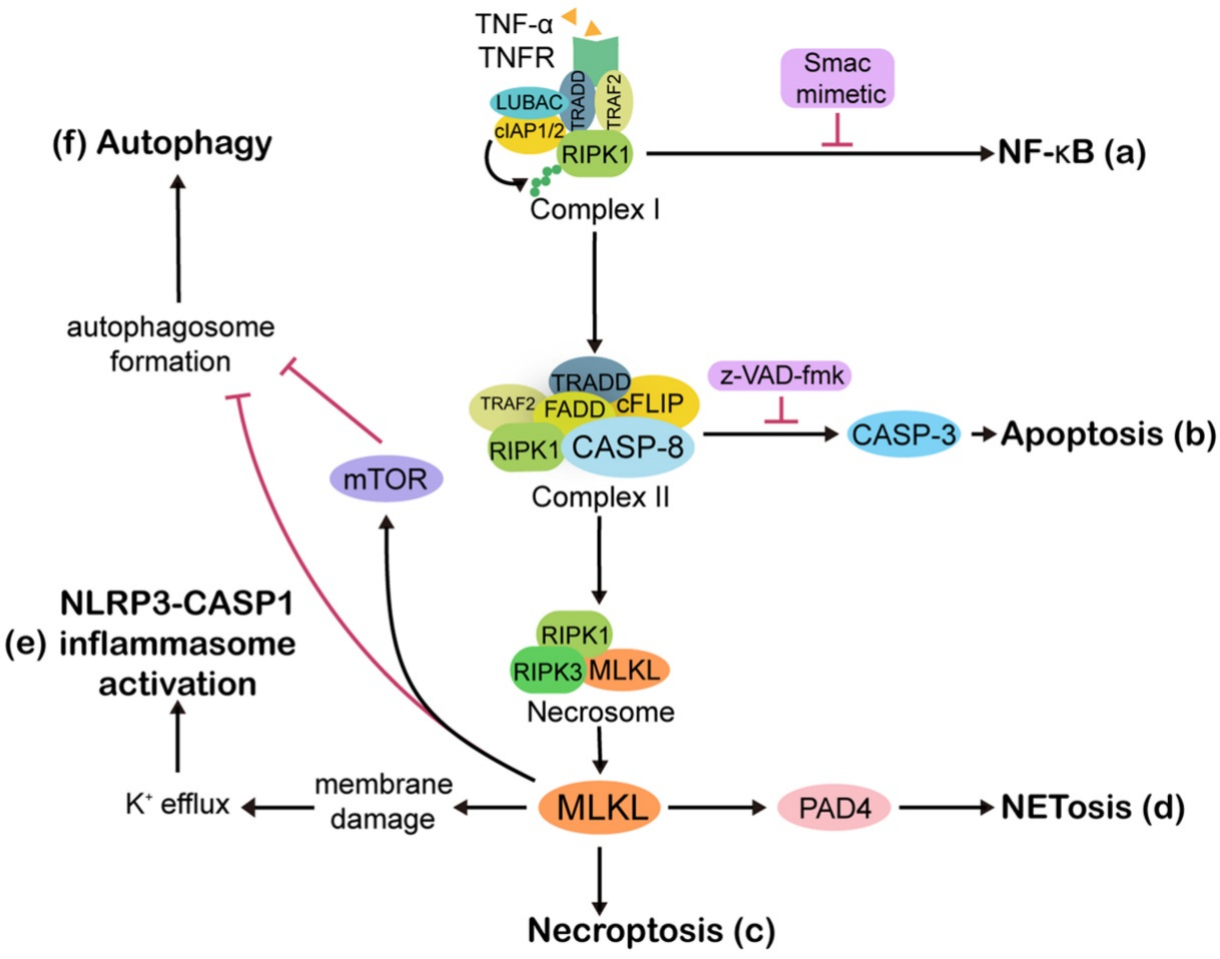
** Overview of the molecular pathways involved in necroptosis.** Ligation of tumor necrosis factor receptor 1 (TNFR) by TNF-α rapidly leads to the formation of complex I, which is made up of TNFR, TNF-associated death domain (TRADD), TNFR-associated factor 2 (TRAF2), receptor interacting protein kinases 1 (RIPK1), cellular inhibitor of apoptosis 1/2 (cIAP1/2), and linear ubiquitin chain assembly complex (LUBAC). Ubiquitin linkages on RIPK1 are decorated by cIAP1/2, followed by NF-κB activation and upregulation of anti-apoptotic proteins (**a**). Once NF-κB is blocked by Smac mimetic, complex I shifts into cytoplasmic complex II, which is comprised of TRADD, TRAF2, RIPK1, Fas associated via death domain (FADD), caspase 8 (CASP8), and cellular FLICE-like inhibitory protein (cFLIP). The cell then moves towards apoptosis (**b**). If CASP8 is compromised by z-VAD-fmk, then RIPK1 and receptor interacting protein kinases 3 (RIPK3) are activated and form a necrosome, facilitating necroptosis (**c**). In some contexts, the necroptosis executioner mixed lineage kinase domain-like protein (MLKL) activates peptidylarginine deiminase 4 (PAD4)-dependent NETosis (**d**). Although there is no direct association between necroptosis and pyroptosis, MLKL can activate pyrin domain-containing 3 (NLRP3)-CASP1 inflammasomes. MLKL causes K^+^ efflux, and then NLRP3 inflammasomes are activated by high-low intracellular K^+^ levels (**e**). Active MLKL inhibits autophagy flux through mammalian target of rapamycin (mTOR)-dependent signaling or by directly suppressing the formation of autophagosomes (**f**). Abbreviations: CASP3, caspase 3; CASP1, caspase 1.

Phenotypically, apoptosis is characterized by nuclear shrinkage, plasma membrane blebbing, chromatin condensation, and regular DNA fragmentation 45, whereas, necroptotic cells show cell swelling and plasma membrane damage, resulting in cell lysis and a release of damage-associated molecular patterns (DAMPs) 46. As RCD pathways, both extrinsic apoptosis and necroptosis can be initiated by common death receptor ligation, among which the tumor necrosis factor receptor (TNFR) is the most widely studied (Figure 2). Ligation of the TNFR promotes the assembly of a dynamic multi-protein complex at the cytoplasmic side of the receptor, which is referred to as “TNFR complex I” and contains TNF-associated death domain (TRADD), TNFR-associated factor 2 (TRAF2), receptor-interacting protein kinases 1 (RIPK1), cellular inhibitor of apoptosis 1 /2 (cIAP1/2), and linear ubiquitin chain assembly complex (LUBAC) 47. Within complex I, cIAP1/2-induced RIPK1 ubiquitination is upstream of nuclear factor-κB (NF-κB) nuclear translocation and is followed by increased expression of a series of pro-inflammatory cytokines and products of anti-apoptotic endogenous proteins, such as cellular FLICE-like inhibitory protein (cFLIP) and cIAP1/2 48-51.

When the complex I-driven NF-κB signaling is blocked by Smac mimetic, cytoplasmic complex II, which consists of TRADD, TRAF2, RIPK1, Fas associated via death domain (FADD), CASP8, and cFLIP, exerts pro-apoptotic functions through CASP8 dimerization and activation 52. If CASP8 or its adaptor protein FADD is compromised, RIPK1 and receptor interacting protein kinases 3 (RIPK3) activation will lead to amyloid necrosomes formation, followed by MLKL oligomerization and translocation to the cell membrane, thus steering cell death from apoptosis towards necroptosis 9, 53, 54. Treating cells with a mixture composed of TNF-α, a Smac mimetic that degrades cIAPs, and the caspase inhibitor z-VAD-fmk is one of the most common methods to induce necroptosis in *in vitro* experiments 55, 56.

Furthermore, necroptosis can be triggered by intricate pathways. For example, viral RNAs can be sensed by DNA-dependent activator of interferon regulatory factors (DAI), and this elicits necroptosis through an interaction between DAI and RIPK3 57. Similarly, Toll-like receptor 3 (TLR3) and Toll-like receptor 4 (TLR4) detect viral RNAs and lipopolysaccharide (LPS) from bacterium, respectively, and this is followed by activation of RIPK3 via the TIR domain-containing adapter-inducing interferon-β (TRIF) 58. Whatever the upstream signaling may be, it is clear that activation of RIPK3 and phosphorylation of MLKL are central events in necroptosis 59-61.

## MLKL functions beyond necroptosis

In the past decade, MLKL has been considered perhaps the only substrate of RIPK3 that can effectively execute necroptosis. Hence, it may represent a molecular target for anti-inflammation and cancer therapies. More recently, its functions other than necroptosis have been gradually recognized. Neglecting the novel functions of MLKL could potentially lead to serious side-effects if MLKL inhibitors are used for the treatment of inflammation or cancer in humans. In addition, because MLKL is involved in many types of diseases (Table 1), a better of understanding of all the functions of MLKL will yield insights for developing control strategies in numerous other physiological and pathological processes.

**Figure 3:**
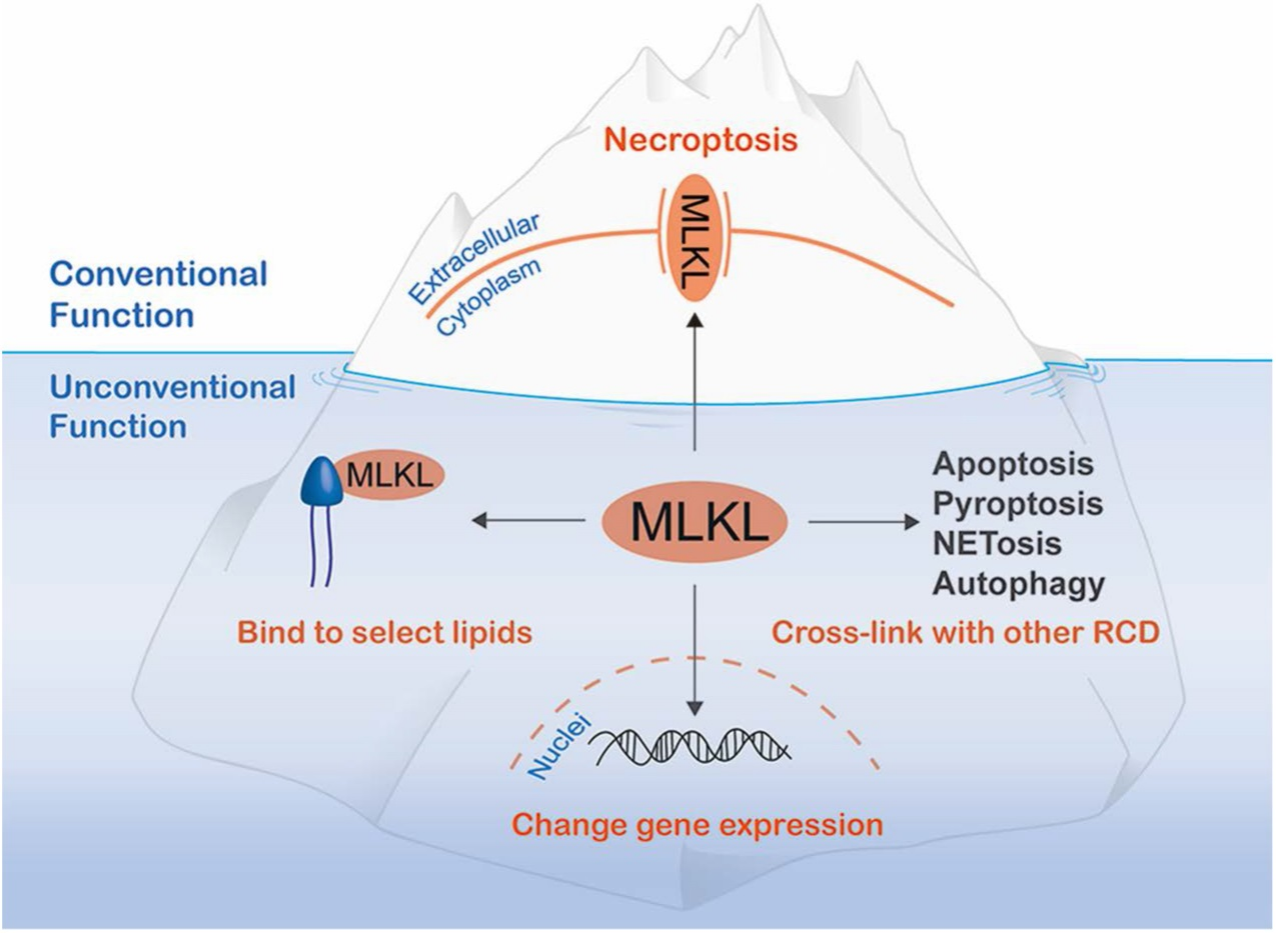
** Additional MLKL functions below sea level.** In addition to its well-known function as a molecule that disrupts plasma membrane integrity to exert necroptosis, MLKL has non-canonical functions that can be divided into the three categories: cross-linking with other regulated cell death (RCD) mediators, translocating to the nucleus to alter gene expression and binding to select lipids to damage bacteria or inhibit a specific metabolic process.

To our knowledge, functions of MLKL beyond serving as the executioner of necroptosis can be attributed to the following three aspects: interaction with other RCD pathways; regulation of gene expression; and combining with lipids to block specific processes (Figure 3). Of note, interaction with other RCD pathways often requires necroptosis. However, the other functions of MLKL can be realized without the risk of necrotic death.

### Non-necroptosis functions of MLKL accompanying necroptosis

Necroptosis does not operate in isolation as is often described in schematic models. In fact, it might affect other RCD or cell death-related pathways, and other RCD pathways also regulate necroptosis 62. This cross-linkages among cell death pathways not only indicates the complexity of RCD but also shows that MLKL participates in many pathways in addition to necroptosis activation (Figure 2).

### Cross-linkages with other RCD

With respect to the cross-linkages among RCD, the linkage between the above-mentioned apoptosis and necroptosis has been most broadly studied, but many other linkages are implicated. In contrast to the opposing relationship between apoptosis and necroptosis, the relationship between necroptosis and other RCD prefers to be synergistic. For example, necroptosis is the basis of NETosis in some situations. NETosis is a type of cell death characterized by neutrophil extracellular trap (NET) formation. Phorbol-12-myristate-13-acetate (PMA) can stimulate NET generation independently of RIPK3 and MLKL signaling 63, 64. In contrast, upon antineutrophil cytoplasmic antibody stimulation or respiratory syncytial virus infection, NET formation occurs via necroptosis 65, 66. Necroptotic NET formation requires the activation of peptidylarginine deiminase 4 (PAD4) 64, 66, and necroptotic NETs located on the membrane are surrounded by MLKL 64. Similar to necroptosis, pyroptosis is also a form of lytic cell death that is triggered by nucleotide-binding oligomerization domain (NOD)-like receptor protein 3 (NLRP3) inflammasome activation and gasdermin D (GSDMD) cleavage. No direct connection has been reported between necroptosis and pyroptosis. However, MLKL can activate NLRP3-caspase 1 (CASP1) inflammasome and interleukin (IL)-1β secretion as a consequence of necroptosis signaling, but before cell lysis. In this case, GSDMD, the pore-forming CASP1 substrate, is dispensable for IL-1β release 67. However, another study found that IL-1β is secreted simultaneously from the dying cells. Further, a MLKL deficiency can reduce the LPS-induced release of IL-1β from LPS-stimulated A20-deficient macrophages. An *in vivo* study reported that necroptosis contributed to inflammasome-associated arthritis and this effect could be inhibited by zinc finger 7 of A20 68. These findings hinted at the possibility that different RCD can co-occur in some cases. And it is difficult to clearly define which type of cell death is predominant in these cases. Regardless, the expression of MLKL and the subsequent cell lysis are key determinants in the co-occurrence of the RCD. Therefore, targeting MLKL might constitute a new approach for therapeutic intervention of relevant diseases such as arthritis.

#### Cross-linkages with cell death-related pathways

Moreover, necroptosis can inhibit pro-survival signaling pathways to promote cell death. Autophagy is a lysosomal digestion and recycling process for cellular contents that promotes cell survival 69, 70. Macroautophagy, the best-characterized form of autophagy, requires the fusion of an autophagosome and lysosome to form an autolysosome. However, necroptosis might inhibit autolysosomal function by compromising lysosomal membrane integrity, subsequently blocking autophagy flux 71. In this context, neither AMP-activated protein kinase (AMPK; autophagy activator) nor the mammalian target of rapamycin (mTOR; autophagy inhibitor) activity is affected by necroptosis. However, when cells are exposed to oxidized low-density lipoprotein, overexpressed MLKL can inhibit autophagy mainly via activation of the mTOR-dependent signaling pathway 72. Thus, necroptotic-mediated regulation of autophagy may vary, depending on the stimulus applied.

### Necroptosis-independent functions of MLKL

#### Regulation of gene expression

Cytoplasmic membrane translocation of cytoplasmic MLKL is necessary for the induction of necroptosis. In addition to cytoplasmic membrane translocation, Yoon et al. found that MLKL also translocates to the nucleus before cell death in necroptosis. Three-dimensional analysis of immunocytochemistry showed that the translocated MLKL exists within the nuclei instead of associating with the nuclear membrane. Of note, the deadly effector function of MLKL is independent of the translocation, and the pharmacological blocking of necroptosis by necrosulfonamide (NSA) has no effect on the nuclear translocation of MLKL 73. Therefore, the nuclear translocation of MLKL might serve some novel functions instead of inducing necroptosis. Given that MLKL does not have a direct DNA or RNA binding domain, therefore MLKL might not directly regulate gene expression. It needs to cooperate with other molecules to exert its regulatory functions. A recent study revealed that MLKL can interact with RNA-binding motif protein 6 (RBM6) to promote expression of adhesion molecules intercellular adhesion molecule-1 (ICAM-1), vascular cell adhesion molecule-1 (VCAM-1), and E-selectin in endothelial cells. Additionally, nuclear translocation of the MLKL-RBM6 complex promotes expression of these molecules by increasing mRNA stability 74. This study confirmed the hypothesis that MLKL can influence gene expression in the nucleus.

Inducing apoptosis is considered to be a promising approach to cancer treatment. Chelerythrine (CHE), a natural benzo[c]phenanthridine alkaloid, can induce apoptosis in tumor cells by promoting ROS production 75, 76. With ROS generation, MLKL translocates from the cytoplasm to the nuclei, facilitating CHE-promoted apoptosis 76. In this case, neither oligomerization nor membrane translocation of MLKL occurs after treatment with CHE. These findings show that MLKL, besides mediating necroptosis, may also contribute to apoptosis via nuclear translocation. Further studies are needed to reveal the nuclear function of MLKL and confirm the necessity of MLKL nuclear translocation in apoptosis.

Additionally, another study reported that depletion of MLKL severely compromises invasion of the nasopharyngeal carcinoma cells accompanied by the reverse of epithelial-mesenchymal transition (EMT). Although the specific mechanism is not clear, it should be noted that MLKL depletion leads to downregulation of 8 hub genes (*VIM, EGFR, JUN, CD44, SPP1, FGF13, PLAU,* and* MMP1*) and upregulation of 2 hub genes (*CHD1* and* MMP9*) 14. However, another study found no difference in lung metastasis when wild type or MLKL knockout breast cancer cells (MVT-1) were injected intravenously 77. This means that MLKL might function in tumor metastasis via regulation of gene expression only in specific tumor cell types. Such cell specificity is not surprising given that tumor metastasis is regulated by numerous proteins 78-80. In particular, the determination of kinases or proteins that activate this non-necroptotic aids in elucidating the cell type-specific manner.

Many cancer cells generally downregulate the expression of necroptotic factors to evade necroptosis and survive 81-83. This suggests that low expression of MLKL may contribute to both tumor survival and regulate gene expression, thus promoting metastasis. Whereas, if MLKL expression is upregulated dramatically, necroptosis may also be induced in cancer cells, which results in cell death. For example, shikonin, a Chinese herbal medicine extract, can trigger necroptosis in nasopharyngeal carcinoma cells via upregulation of MLKL expression 84. Also of note, the inflammatory response can be irritated by necroptosis in the body, which may provide a microenvironment suitable for cancer progression 85. Thus, MLKL is a potential target for anti-tumor treatments but upregulation of MLKL is not a one-size-fits-all approach to cancer.

#### Blocking specific processes via the affinity of MLKL for select phospholipids and cardiolipin

Similar to perforation in necroptosis, MLKL may exert its necroptosis-independent functions via affinity for select molecules, such as phosphatidylinositol, cardiolipin, sulfatide, and so on. For example, p-MLKL can bind to PI(4,5)P_2_ to inhibit insulin-stimulated PI(3,4,5)P_3_ production at the plasma membrane in hepatocytes 86. This process leads to the pathophysiology of hepatic insulin resistance and type 2 diabetes. Intriguingly, MLKL does not affect hepatic inflammation and liver cell death under obese conditions. Murine models of non-alcoholic fatty liver disease provide another example that MLKL translocates to autophagosomes, a spherical structure with double-layer membranes, prior to reaching the plasma membrane and blocks the autophagic flux resulting in liver injury 87. The necroptosis-independent functions of MLKL seem to rely on the interactions between 4HB and other molecules.

Necroptosis plays a positive role in at least two ways. First, it limits pathogen spread by preventing the host cell from becoming a pathogen factory, and second, it galvanizes the adaptive immune response via the release of debris into the extracellular space 88. Both of these pathways involve cell destruction, leading to cell death. However, a recently study found that infection by *Listeria monocytogenes*, a gram-positive enteropathogenic bacteria, can activate the RIPK3-MLKL pathway in the infected cell without causing necroptosis 11. Additionally, the monomer p-MLKL can bind to cardiolipin of *Listeria*, which is immediately followed by decreased *Listeria* replication. Of note, cardiolipin is more abundant on the *Listeria* cell membrane compared with the outer membranes of gram-negative bacteria, which perhaps explains why p-MLKL does not associate with *Salmonella typhimurium* 11. Intriguingly, although MLKL can be activated by RIPK3 in *Listeria*-infected intestine epithelial cells, no MLKL oligomer formation and membranal translocation occurs in these cells 11. Considering the fact that pharmacologic or genetic targeting of TAM kinases has no impact on the membranal translocation of MLKL, other mechanisms, apart from inhibition of TAM kinase, must be responsible for this phenomenon 28.

As for the MLKL-sulfatide interaction, it plays an important role in demyelination, not only in the central nervous system (CNS), but also in the peripheral nervous system (PNS). Sulfatide is crucial to the maintenance of an intact myelin structure 89. The myelin sheath is a lipid-rich substance formed by oligodendrocytes in the CNS and by Schwann cells in the PNS 90. Multi-layered specialized plasma membranes protect the axons and speed the transmission of electrical impulses along myelinated axons. In the CNS, multiple sclerosis (MS), as a demyelination disease, is coordinated by necroptosis-independent MLKL 13. Lack of MLKL protein delays chemical-induced (cuprizone) demyelination of MS, whereas, intriguingly, a recent study in two siblings reported that a variant in *MLKL* led to a deficiency of MLKL resulting in a disorder that shares clinical features with primary progressive MS and other neurodegenerative diseases 91. In the PNS, successful regeneration of myelinated nerves requires the breakdown and clearance of myelin. A study showed that injury-induced phosphorylation of serine 441 in mouse MLKL targets the myelin sheath membrane of Schwann cells to promote demyelination and regeneration of axons 12. Because the necroptosis- and nerve injury-associated phosphorylation pathways represent non-overlapping pathways, it is clear that different functions of MLKL result from different phosphorylation sites via different upstream kinases.

Existing evidence shows that MLKL and/or p-MLKL can be released within the extracellular vesicles (EVs) 92, 93, suggesting that the above-mentioned functions of MLKL may be regulated by endosomal trafficking and generation of EVs. This release appears to inhibit cell death. For example, syncytiotrophoblasts release EVs that contain Hsp 70, misfolded proteins, and MLKL, to eliminate intracellular increased toxins 94. However, these dangerous/toxic EVs can lead to maternal endothelial cell activation, causing preeclampsia. Interestingly, MLKL is involved in endosomal trafficking and the generation of small EVs by interaction with endosomal sorting complexes required for transport (ESCRT) proteins and flotillins 92. MLKL facilitates the generation of EVs, and this function is independent of phosphorylation but can be enhanced by it. MLKL also contributes to direct budding of the damaged plasma membranes, which results in the generation of phosphatidylserine positive and p-MLKL containing EVs 93. This process also depends on the function of ESCRT-III activity. ESCRT-III components can sustain the survival of cells when MLKL activation is subsequently halted.

The current evidence from these studies suggests both the C-terminal pseudokinase domain and the N-terminal 4HB domain are the bases of non-necroptosis functions of MLKL. On the one hand, the nuclear localization signal motif-containing pseudokinase domain of MLKL is required for nuclear shuttling 73, but on the other hand, clear evidence indicates that 4HB is a required region of MLKL in necroptosis 95. Currently, most studies focusing on necroptosis-independent functions of MLKL suggest that these functions are also dependent on MLKL to phosphatidylinositol, cardiolipin, sulfatide, and so on. However, the reasons for MLKL translocation to specific structures instead of the plasma membrane have not yet been clarified. Furthermore, these studies also raise other fascinating questions as do all other interesting findings 11, 12, 87. For example, why does p-MLKL not oligomerize and damage the membrane structure in some contexts? Apart from RIPK3, what other proteins can phosphorylate MLKL? Apart from the known phosphorylation sites, are there several other phosphorylation sites for which the functional role is unclear? Furthermore, at present, no study has confirmed the regulation mode of MLKL in the same way as has been achieved in other pseudokinase situations. More experiments are therefore needed to investigate whether MLKL can serve as a mediator through the regulation of specific kinase function 96.

## MLKL inhibitors

Increasing evidence shows that necroptosis contributes to the pathology of many severe diseases 97, especially inflammation and cancer, and numerous researchers have attempted to develop drugs to block MLKL function. A full understanding of the mechanism of action of MLKL inhibitors may be helpful in devising more potent drug therapies. And more importantly, it might also be speculated whether these currently available agents can inhibit non-necroptotic functions of MLKL. The first well-defined MLKL inhibitor is compound 1. Compound 1 (an ATP mimetic also named GW806742X) interacts with the nucleotide-binding site in the mouse MLKL pseudokinase domain, to exposure the MLKL activation loop. This interaction inhibits necroptosis by suppressing MLKL conformational change 95. Of note, compound 1 can affect cell viability at high concentrations and may have off-target activity against RIPK1, RIPK3, and vascular endothelial growth factor receptor 2 95, 98, 99. A later study showed that the binding of compound 1 to the ATP-pocket of MLKL could not rescue cells from necroptosis, and the effect of compound 1 on necroptosis might be due to its non-specific binding to RIPK1 and other kinases 100. Thus, considering the fact that different kinases activate different functions of MLKL, compound 1, as a potent pharmacological inhibitor of necroptosis, might be unable to completely inhibit the non-necroptotic function of MLKL. Unlike compound 1 described above, necroptosis-blocking compound 1 (NBC1) is another small molecule that conjugates with Hsp70 to inhibit MLKL polymerization, instead of directly acting on MLKL 42. Up to date, no study has yet reported the effects of Hsp (e.g., Hsp70 and Hsp90) on non-necroptotic functions of MLKL. Therefore, it is difficult to speculate that whether NBC1 can act on the non-necroptotic functions.

To date, NSA is the most widely used MLKL inhibitor in experimental research. NSA can directly target the Cys86 residue in the N-terminal domain and block necroptosis in human cells 20, 25, 101. Furthermore, NSA can cross-link Cys86 of human MLKL to Cys32 of thioredoxin-1, which then suppresses necroptosis by inhibiting disulfide bond formation between monomeric MLKL 102. In addition to NSA, compound TC13172 is another MLKL inhibitor that induces covalent binding at Cys86 of MLKL 99. Nevertheless, NSA and TC13172 do not inhibit necroptosis of mouse cells, because position 86 in mouse MLKL is a tryptophan residue, instead of a cysteine residue 20, 99. This precludes the use of these inhibitors in pre-clinical mouse models. Recently, *Petrie* et al. developed inhibitory monobodies, synthetic binding proteins, that potently block necroptosis 103. Both NSA and inhibitory monobodies only block MLKL translocation to membranes, but do not prevent human MLKL phosphorylation or oligomerization 36, 103, 104. As we stated earlier, MLKL is to elicit non-necroptotic functions by employing a cytomembrane translocation-independent mechanism in most cases. The increase in intracellular MLKL may facilitate the regulation of gene expression and the affinity of MLKL for select phospholipids and cardiolipin to some extent. Therefore, rather than contributing to inhibit non-necroptotic functions, MLKL inhibitors, which aim to halt necroptosis, may instead promote these functions.

Even though existing MLKL inhibitors can effectively inhibit necroptosis, their inhibitory abilities for most of the non-necroptotic functions remain undetermined. The investigation of currently available MLKL inhibitors will be crucial to determining their effectiveness in regulating non-necroptotic functions. Besides, the functional complexity of MLKL requires further development of novel inhibitors. There are several approaches that could be adopted to inhibit non-necroptotic functions of MLKL: (a) blocking MLKL activation; (b) occupying MLKL-binding sites on phosphatidylinositol and cardiolipin; or (c) inhibiting intracellular movement of activated MLKL. Considering the beneficial role of MLKL in certain cases, future experiments are also needed to develop an inhibitor of necroptosis that does not compromise the beneficial functions of MLKL.

## Conclusions

Most studies of MLKL to date have been restricted to necroptosis, and its non-necroptotic functions have received scant attention. The current review shows that MLKL is integrated into a network of pleiotropic functions. As a consequence, how exactly MLKL exerts its non-necroptotic functions is not yet clear. However, based on the existing literature, we can speculate that some of the non-necroptotic functions of MLKL, are also dependent upon its affinity for specific membrane structures. Likewise, MLKL can regulate other RCD pathways and even the expression of some genes. We are optimistic that further studies will provide insight avoiding the side effects of MLKL inhibitors in future applications and for controlling diseases through regulation of non-necroptotic functions of MLKL.

## Figures and Tables

**Table 1:** Reported non-necroptosis functions of MLKL and its role in several specific diseases

Disease	Cell type	RIPK3 activation	MLKL phosphorylation	MLKL oligomerization	Phenotype	Function	Reference
Type 2 diabetes	Hepatocyte	+	+	+	Insulin resistance	Inhibiting PIP3 production	86
Non-alcoholic fatty liver disease	Hepatocyte	-	+	+	Hepatocellular injury	Suppressing autophagic flux	87
*Listeria monocytogenes* infection	Intestine epithelial cell	+	+	-	Slow-multiplying bacterial states of *Listeria* in epithelial cells	Impinging on bacterium to disrupt their plasma membranes	11
Multiple sclerosis	Oligodendrocyte	-	+	Unknown	Increased demyelination	Unknown	13
Nerve regeneration	Schwann cell	-	+	Unknown	Increased demyelination	Loss of the myelin structure maintenance function of sulfatide	12
Nasopharyngeal carcinoma	Radioresistant nasopharyngeal cell	-	-	Unknown	Irradiation-induced invasion	Promoting epithelial-mesenchymal transition	14
Loss of the pregnancy	Syncytiotrophoblast	-	-	Unknown	Release of dangerous proteins	Facilitating endosomal trafficking and generation of extracellular vesicles	94

## Abbreviations

MLKL: mixed lineage kinase domain-like protein
ACD: accidental cell death
RCD: regulated cell death
CASP 8: caspase 8
4HB: four-helix bundle
TAM kinase: Tyro3, Axl, and Mer kinase
PIPs: phosphatidylinositol phosphates
Hsp90: heat shock protein 90
Hsp70: heat shock protein 70
Bcl-2: B-cell lymphoma 2
BH: Bcl-2 homology
DAMPs: damage-associated molecular patterns
TNFR: tumor necrosis factor receptor
TRADD: TNF-associated death domain
TRAF2: TNFR-associated factor 2
RIPK1: receptor-interacting protein kinases 1
cIAP1/2: cellular inhibitor of apoptosis 1 /2
LUBAC: linear ubiquitin chain assembly complex
NF-κB: nuclear factor-κB
cFLIP: cellular FLICE-like inhibitory protein
FADD: Fas associated via death domain
RIPK3: receptor interacting protein kinases 3
DAI: DNA-dependent activator of interferon regulatory factors
TLR3: Toll-like receptor 3
TLR4: Toll-like receptor 4
TRIF: TIR domain-containing adapter-inducing interferon-β
NET: neutrophil extracellular trap
PMA: phorbol-12-myristate-13-acetate
PAD4: peptidylarginine deiminase 4
NOD: nucleotide-binding oligomerization domain
NLRP3: NOD-like receptor protein 3
GSDMD: gasdermin D
CASP1: caspase 1
AMPK: AMP-activated protein kinase
mTOR: mammalian target of rapamycin
NSA: necrosulfonamide
RBM6: RNA-binding motif protein 6
CHE: chelerythrine
ROS: reactive oxygen species
EMT: epithelial-mesenchymal transition
p-MLKL: phosphorylated MLKL
CNS: central nervous system
PNS: peripheral nervous system
MS: multiple sclerosis
EVs: extracellular vesicles
ESCRT: endosomal sorting complexes required for transport
NBC1: necroptosis-blocking compound 1
